# Weighty Matters: A Real-World Comparison of the Handtevy and Broselow Methods of Prehospital Weight Estimation

**DOI:** 10.1017/S1049023X22001248

**Published:** 2022-10

**Authors:** Chloe Knudsen-Robbins, Phung K. Pham, Kim Zaky, Shelley Brukman, Carl Schultz, Claus Hecht, Kellie Bacon, Maxwell Wickens, Theodore Heyming

**Affiliations:** 1. University of Pittsburgh School of Medicine, Pittsburgh, Pennsylvania USA; 2. Division of Emergency and Transport Medicine, Children’s Hospital Los Angeles, Los Angeles, California USA; 3. Department of Emergency Medicine, Children’s Health of Orange County, Orange, California USA; 4. Orange County Health Care Agency, Santa Ana, California USA; 5. Orange County Fire Authority, Irvine, California USA; 6. Department of Emergency Medicine, University of California, Irvine, California USA

**Keywords:** body weights and measures, pediatric, prehospital emergency care

## Abstract

**Introduction:**

The majority of pediatric medications are dosed according to weight and therefore accurate weight assessment is essential. However, this can be difficult in the unpredictable and peripatetic prehospital care setting, and medication errors are common. The Handtevy method and the Broselow tape are two systems designed to guide Emergency Medical Services (EMS) providers in both pediatric patient weight estimation and medication dosing. The accuracy of the Handtevy method of weight estimation as practiced in the field by EMS has not been previously examined.

**Study Objective:**

The primary objective of this study was to examine the field performance of the Handtevy method and the Broselow tape with respect to prehospital patient weight estimation.

**Methods:**

This was a retrospective chart review of trauma and non-trauma patients transported by EMS to the emergency department (ED) of a quaternary care children’s hospital from January 1, 2021 through June 30, 2021. Demographic data, ED visit information, prehospital weight estimation, and medication dosing were collected and analyzed. Scale-based weight from the ED was used as the standard for comparison.

**Results:**

A total of 509 patients <13 years of age were included in this study. The EMS providers using the Broselow method estimated patient weight to within +/-10% of ED scale weight in 51.3% of patients. When using the Handtevy method, the EMS providers estimated patient weight to within +/-10% of ED scale weight in 43.7% of patients. When comparing the Handtevy versus Broselow method of prehospital weight estimation, there was no significant association between method and categorized weight discrepancy (over, under, or accurate estimates – defined as within 10% of ED scale weight; P = .25) or percent weight discrepancy (P = .75). On average, prehospital weight estimation was 6.33% lower than ED weight with use of the Handtevy method and 6.94% lower with use of the Broselow method.

**Conclusion:**

This study demonstrated no statistically significant difference between the use of the Handtevy or Broselow methods with respect to prehospital weight estimation. While further research is necessary, these results suggest similar field performance of the Broselow and Handtevy methods.

## Introduction

Medication errors in the pediatric patient population are quite prevalent. In the pediatric in-patient setting, Kaushal, et al reported an error rate of approximately 55 errors for every 100 admissions, the majority (28%) being dosing errors, and a potential adverse drug event rate of 10/100 admissions.^1^ Medication errors have been shown to occur more frequently in the emergency department (ED), and error rates rise even further in the prehospital setting to just under 35% for all medications and over 60% for epinephrine.^2–4^ These errors are estimated to affect over 21,000 US children under the age of 11 each year.^4^

Pediatric medication errors have been attributed to a number of causes, including weight-based dosing and the increased number of calculations required for correct dosing and administration.^1^ Many of the complications inherent to pediatric medication dosing are potentiated by the unpredictable nature of the prehospital setting. As pediatric medication dosing is predominantly weight-based, accurate assessment is essential. However, in prehospital care, obtaining reliable weight measurements is not straightforward; it is estimated that approximately 20% of out-of-hospital pediatric weight estimates are not accurate.^5^

Many tools have been developed to assist with pediatric weight estimation, including age-based formulas, length-based tapes, paperboard dosing wheels, and electronic applications.^5^ The Broselow method, which provides a weight estimation based on length as measured using the Broselow tape, is the most commonly used length-based tape. The Broselow method was developed in the 1980s and has been modified and studied fairly extensively in the intervening years, with varying results.^4,6–20^

The Handtevy system, initially developed in 2010 as a length and age-based system, has been adapted and can now be used as an exclusively age-based tool for weight estimation.^21^ There are few studies evaluating the Handtevy system, most of which were published prior to the development of the solely age-based application, however Rappaport, et al recently published a manuscript reporting almost 90% dosing accuracy using the Handtevy Field Guide (accuracy was based on correct use of the field guide/patient age, not patient weight).^12,17,22^ There appear to be no studies in the published literature evaluating the accuracy of the Handtevy weight estimation method in the prehospital setting.

The primary objective of this study was to assess the field performance of the Broselow tape and the Handtevy method with respect to prehospital pediatric patient weight estimations. Secondary objectives included evaluating accuracy of medication dosing, the percentage of patients placed in accurate weight categories using the Broselow method, and comparing ED weight by age to predicted Handtevy weights.

## Methods

This retrospective cohort study included trauma and non-trauma pediatric patients transported via Emergency Medical Services (EMS) to the ED of a Level 2 trauma center/Comprehensive Children’s Receiving Center from January 1, 2021 through June 30, 2021. Data were collected via retrospective chart review of ED records by a team of trained data abstractors using a standardized REDCap (Vanderbilt University; Nashville, Tennessee USA) data collection form. Demographics, EMS provider, prehospital weight estimation, prehospital medication/dose, ED weight, ED intubation status, and ED disposition were collected. Prehospital weight estimation method was based on EMS provider policy and statistics were calculated based on an intention to treat model. Scale-based weight from the ED was used as the standard for comparison.

All patients transported via EMS to the study institution for the duration of the study were included unless they met exclusion criteria. Patients with cerebral palsy and contractures were excluded, as were any interfacility transports (including transports from skilled nursing facilities), patients for whom ED weights were not obtained using a scale, and patients whose charts contained incomplete study data. Abstractors were trained research assistants who followed a standardized operating manual for data abstraction (including instructions regarding where each variable may be found in the chart). Abstractors completed an iterative inter-rater reliability process in which each abstractor abstracted variables for 10 randomly selected charts and any discrepancies were analyzed by TH, PKP, and CKR. Necessary changes to the operating manual identified by this process were completed between iterations. Following complete data abstraction, simple random sampling was used to select 10% of charts for repeat abstraction to assess data entry reliability. REDCap data were screened and cleaned by TH, PKP, and CKR and EMS runs with insufficient or inconsistent data were excluded.

As the intended scope of the Broselow method was predominantly directed towards children <13 years of age, and a majority of studies include subsets of children younger than this, the Handtevy and Broselow comparisons regarding weight estimation and medication dosing was limited to those under 13.^6,12,22,23^ A secondary aim of this study was to compare the Handtevy suggested weight estimation based on age to ED weights. As the Handtevy method is designed for patients ≤13 years, children ≤13 years of age were included in this additional analysis.

In this study, a single EMS agency (Provider “Z”) used the Handtevy method. This provider transports >50% of patients transported via EMS to the study institution. Provider Z transitioned from the Broselow method to the Handtevy method midway through the study, allowing for three months of baseline and three months of comparative data to be collected. Related analyses will subsequently be described as “pre-Handtevy” Z-Provider or “post-transition” Z-Provider. All other EMS agencies employed the Broselow method and will be referred to collectively as “Y-Providers.” The Broselow tape used by EMS in this study was the Broselow 2007 edition, version B; the Handtevy method used in this study was v4.2.7.

Only weight-based EMS-administered medications were considered for this study; these included adenosine, amiodarone, atropine, dextrose, diphenhydramine, epinephrine, fentanyl, hydroxocobalamin, lidocaine, midazolam, morphine, and sodium bicarbonate. Dosing was considered accurate based on ED weight and county policy. Patients transported via out of county EMS providers were excluded from medication analysis. This study was approved by Children’s Health of Orange County (Orange, California USA) In-House Institutional Review Board (#210682).

### Effect Size Calculations

A pair of effect size calculations were conducted using G*Power (version 3.1.9.3; Faul and Erdfelder; Germany), since the total sample size was bounded by the specific evaluation period (January 1, 2021 through June 30, 2021).^24^ The first effect size calculation was conducted under the family of exact tests, in which statistical power was set to 90% with two-tailed alpha at 0.05, and 54% was entered as the base accuracy rate of the Broselow method.^18^ For the effect of the Handtevy method to be statistically significant, its accuracy rate would have to differ from the Broselow method by at least 15% (ie, Handtevy accuracy rate ≥69% or ≤39%).

The second effect size calculation was conducted under the family of t-tests, in which statistical power was set to 90% with two-tailed alpha at 0.05. For the effect of the Handtevy method to be statistically significant, its mean percent weight discrepancy would have to differ from the Broselow method by at least six percent if the accompanying standard deviation is large (eg, standard deviation of 20% for Handtevy mean percent weight discrepancy of five percent).

### Statistical Analysis

Chi-squared test was used to analyze categorized weight discrepancy (over, under, or accurate estimates – defined as within 10% of ED scale weight) and the Broselow/Handtevy methods; independent samples t-test was used to analyze percent weight discrepancy and the Broselow/Handtevy methods. Percent weight discrepancy was calculated as [(EMS weight – ED weight)/ED weight]x100. Negative values signified EMS under-estimation in patient weight, while positive values signified EMS over-estimation of patient weight. Chi-squared test was used to analyze categorized weight discrepancy and Z pre-Handtevy/Z post-transition/Y; three-way between-subjects ANOVA (Handtevy/Broselow methods; non-Hispanic/Hispanic ethnicity; age groups) was used to further analyze percent weight discrepancy. Chi-squared test with Monte Carlo simulation was used to analyze medication dosing (under, over, and accurately dosed based on ED scale weight) and the Broselow/Handtevy methods. Chi-squared test with Monte Carlo simulation was also used to analyze medication type and dosing. Statistical analyses were performed using IBM SPSS Statistics, version 26 (IBM Corp.; Armonk, New York USA).

## Results

### Patient Characteristics

A total of 509 EMS transports were analyzed to address the primary objective of this study. Overall characteristics for this sample of patients <13 years of age were as follows: 265 (52.1%) were male; 297 (58.3%) were Hispanic; 348 (68.4%) had public insurance; five (1%) were intubated in the ED; 369 (72.5%) were discharged home; 77 (15.1%) were admitted to the general floor or to surgery; 23 (4.5%) were admitted to the pediatric intensive care unit (PICU); eight (1.6%) were transferred to an external medical facility; 31 (6.1%) were admitted to this institution’s in-patient mental health unit or transferred to an external mental health facility; and one (0.2%) died. Further data including frequencies by Provider-Z pre-Handtevy, Provider-Z post-transition, and Providers-Y may be found in Table 1.

**Table 1.** Patient Characteristics by EMS Provider and Weight Estimation Method

Patient Characteristic	Handtevy, Z-Provider, Age ≤ 13y N = 235 n (%)	Handtevy, Z-Provider, Age < 13y, N = 197 n (%)	Broselow, Z-Provider, Age < 13y N = 111 n (%)	Broselow, Y-Providers, Age < 13y N = 247 n (%)	Broselow, All Providers, Age < 13y N = 312 n (%)	Handtevy & Broselow, All Providers, Age < 13y N = 509 n (%)
*Biological Sex*						
Male	136 (57.9%)	114 (57.9%)	54 (48.6%)	125 (50.6%)	151 (48.4%)	265 (52.1%)
Female	99 (42.1%)	83 (42.1%)	57 (51.4%)	122 (49.4%)	161 (51.6%)	244 (47.9%)
*Ethnicity*						
Hispanic	150 (63.8%)	130 (66.0%)	64 (57.7%)	126 (51.0%)	167 (53.5%)	297 (58.3%)
Non-Hispanic	84 (35.7%)	67 (33.5%)	45 (40.5%)	120 (48.6%)	142 (45.5%)	208 (40.9%)
Missing Data	1 (0.4%)	1 (0.5%)	2 (1.8%)	1 (0.4%)	3 (1.0%)	4 (0.8%)
*Race*						
White/Caucasian	118 (50.2%)	102 (51.8%)	56 (50.5%)	148 (60.0%)	178 (57.1%)	280 (55.0%)
Asian or Pacific Islander	34 (14.5%)	26 (13.2%)	16 (14.4%)	21 (8.5%)	33 (10.6%)	59 (11.6%)
Black/African American	6 (2.6%)	6 (3.3%)	2 (1.8%)	10 (4.0%)	10 (3.2%)	16 (3.1%)
Another Identity or Bi-/Multi-Racial	75 (31.9%)	61 (31.0%)	36 (32.4%)	68 (27.5%)	90 (28.8%)	151 (29.7%)
Missing Data	2 (0.9%)	2 (1.0%)	1 (0.9%)	0 (0.0%)	1 (0.3%)	3 (0.6%)
*Intubation*						
No	230 (97.9%)	196 (99.5%)	110 (99.1%)	241 (97.6%)	308 (98.7%)	504 (99.0%)
Yes	5 (2.1%)	1 (0.5%)	1 (0.9%)	6 (2.4%)	4 (1.3%)	5 (1.0%)
*ED Disposition*						
Discharged Home	174 (74.0%)	155 (78.7%)	84 (75.7%)	157 (63.6%)	214 (68.6%)	369 (72.5%)
Admitted to Medical/Surgical Floor	36 (15.3%)	26 (13.2%)	19 (17.1%)	41 (16.6%)	51 (16.3%)	77 (15.1%)
Admitted to PICU	13 (5.5%)	6 (3.0%)	3 (2.7%)	23 (9.3%)	17 (5.4%)	23 (4.5%)
Transferred to Mental Health Facility	6 (2.6%)	5 (2.5%)	3 (2.7%)	23 (9.3%)	26 (8.3%)	31 (6.1%)
Transferred to Outside Medical Facility	6 (2.6%)	5 (2.5%)	2 (1.8%)	2 (0.8%)	3 (1.0%)	8 (1.6%)
Expired	0 (0.0%)	0 (0.0%)	0 (0.0%)	1 (0.4%)	0 (0.0%)	1 (0.2%)
*Health Insurance*						
Public Insurance	167 (71.1%)	144 (73.1%)	78 (70.3%)	146 (59.1%)	204 (65.4%)	348 (68.4%)
Private Insurance	65 (27.7%)	50 (25.4%)	33 (29.7%)	96 (38.9%)	105 (33.7%)	155 (30.4%)
Military Insurance	3 (1.3%)	3 (1.5%)	0 (0.0%)	2 (0.8%)	2 (0.6%)	5 (1.0%)
Self-Pay	0 (0.0%)	0 (0.0%)	0 (0.0%)	3 (1.2%)	1 (0.3%)	1 (0.2%)

Abbreviations: ED, emergency department; PICU, pediatric intensive care unit.

### Prehospital Weight Estimation (Table 2a and Table 2b)

**Table 2a.** Weight Discrepancy by Weight Estimation Method

Weight Estimation Method (n)	Categorized Weight Discrepancy			Percent Weight Discrepancy ^a^				
	Estimation Within +/-10% n (%)	Under-Estimation n (%)	Over-Estimation n (%)	Mean	SD	Median	Range	95% CI of the Mean
Handtevy (197)	86 (43.7%)	79 (40.1%)	32 (16.2%)	−6.33%	23.65	−5.56	−97.24 to 69.97	−9.65 −3.00
Broselow (312)	160 (51.3%)	107 (34.3%)	45 (14.4%)	−6.94%	19.9	−4.82	−67.63 to 62.98	−9.16 −4.72
*Total (509)*	*247 (48.5%)*	*186* *(36.5%)*	*77* *(15.0%)*	*-6.70%*	*21.41*	*-5.04*	*-97.24 to 69.97*	*-8.57 −4.84*

Abbreviations: ED, emergency department; EMS, Emergency Medical Services.

a Calculated as [(EMS weight – ED weight)/ED weight]x100. When the value is negative, EMS under-estimated; when the value is positive, EMS over-estimated.

**Table 2b.** Weight Discrepancy by Agency and Weight Estimation Method

Agency/Method (n)	Categorized Weight Discrepancy			Percent Weight Discrepancy ^a^				
	Estimation Within +/-10% n (%)	Under-Estimation n (%)	Over-Estimation n (%)	Mean	SD	Median	Range	95% CI of the Mean
Handtevy by Z-Provider (197)	86 (43.7%)	79 (40.1%)	32 (16.2%)	−6.33%	23.65	−5.56	−97.24 to 69.97	−9.65 −3.00
Broselow by Z-Provider (104)	50 (48.1%)	37 (35.6%)	17 (16.3%)	−7.16%	20.46	−5.06	−59.52 to 62.98	−11.14 −3.18
Broselow by Y-Providers (208)	110 (52.9%)	70 (33.7%)	28 (13.5%)	−6.83%	19.67	−4.75	−67.63 to 54.37	−9.52 −4.14
*Total (509)*	*247 (48.5%)*	*186 (36.5%)*	*77 (15.0%)*	*-6.70%*	*21.41*	*-5.04*	*-97.24 to 69.97*	*-8.57 −4.84*

Abbreviations: ED, emergency department; EMS, Emergency Medical Services.

a Calculated as [(EMS weight – ED weight)/ED weight]x100. When the value is negative, EMS under-estimated; when the value is positive, EMS over-estimated.

The EMS providers using the Broselow method estimated patient weight within +/-10% of ED scale weight in 51.3% of patients (ie, prehospital weight was not over or under ED scale weight by more than 10%). When using the Handtevy method, EMS providers estimated patient weight to within +/-10% of ED scale weight in 43.7% of patients. When comparing the Handtevy versus Broselow method of prehospital weight estimation, there was no significant association between method and categorized weight discrepancy (over, under, or accurate estimates – defined as within 10% of ED scale weight; P = .25), as well as no significant difference in percent weight discrepancy (P = .75). On average, prehospital weight estimations were 6.7% lower than ED scale weight for the same patient.

**Figure 1.**
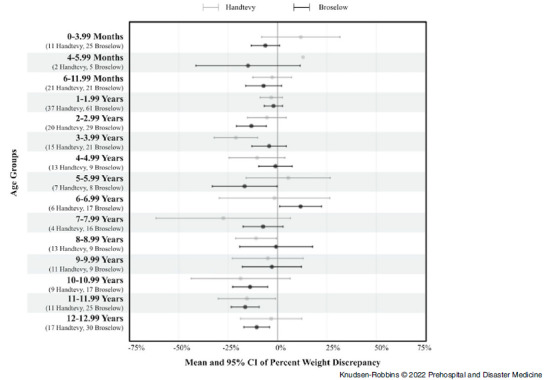
Mean and 95% CI of Percent Weight Discrepancy by Age Groups. Note: Given only two Handtevy EMS runs in the 4-5.99mo age group, no confidence interval was outputted. Abbreviation: EMS, Emergency Medical Services.

**Figure 2.**
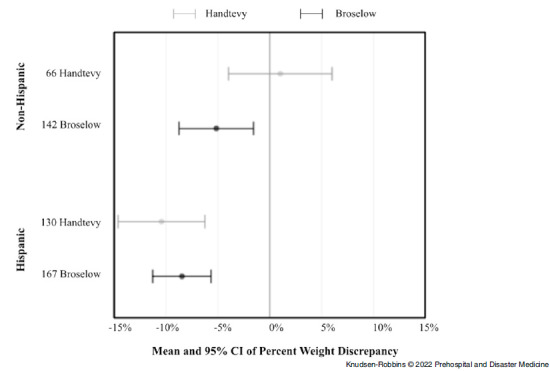
Mean and 95% CI of Percent Weight Discrepancy by Ethnicity. Note: Ethnicity data missing from four EMS runs. Abbreviation: EMS, Emergency Medical Services.

In addition, when stratifying by Provider-Z pre-Handtevy, Provider-Z post-transition, and Y-Providers, there was no association with categorized weight discrepancy (P = .46). When percent weight discrepancy was further analyzed, significant interactions were found of the Handtevy/Broselow methods with non-Hispanic/Hispanic ethnicity (P = .005) and with age groups (P = .02). Data visualizations show substantial percent weight discrepancies from these interactive effects (Figure 1 and Figure 2). Handtevy weight estimations were noticeably less discrepant for non-Hispanic patients compared to Hispanic patients. The Broselow method tended to under-estimate weight for both non-Hispanic and Hispanic patients.

### Medication Dosing

*Pediatric Intensive Care Unit (PICU)*—There were only 22 EMS runs in which the medications of research interest were administered (n = 24 discrete medications); 10 patients received midazolam, four received fentanyl, three patients received diphenhydramine, and three received epinephrine. Two patients received both diphenhydramine and epinephrine. There was no significant association between medication dosing (under, over, or accurate dosing) and use of either the Broselow or Handtevy weight estimation methods (P = .85; Table 3a). Additionally, there was no significant association between medication type and dosing (P = .63; Table 3b).

**Table 3a.** Medication Dosing Accuracy by Weight Estimation Method

Weight Estimation Method (n)	Under Dosed n (%)	Over Dosed n (%)	Accurately Dosed n (%)
Handtevy (8)	1 (12.5%)	2 (25.0%)	5 (62.5%)
Broselow (16)	3 (18.75%)	6 (37.5%)	7 (43.75%)
*Total (24)*	*4 (16.7%)*	*8 (33.3%)*	*12 (50.0%)*

**Table 3b.** Medication Dosing Accuracy by Medication

Medication (n)	Under Dosed n (%)	Over Dosed n (%)	Accurately Dosed n (%)
Diphenhydramine (5)	2 (40%)	1 (20%)	2 (40%)
Epinephrine (5)	1 (20%)	1 (20%)	3 (60%)
Fentanyl (4)	0 (0%)	1 (25%)	3 (75%)
Midazolam (10)	1 (10%)	5 (50%)	4 (40%)
*Total (24)*	*4 (16.7%)*	*8 (33.3%)*	*12 (50.0%)*

*Broselow (*Table 4 *)—*Among patients for whom the Broselow method of estimation was used, 42.7% had estimated weights which placed them into the correct Broselow category according to their ED weight.

**Table 4.** ED Scale Weights Compared to Broselow Predictions for Patients <13 Years of Age ^a^

Broselow Zone	Predicted Weight Range	ED Scale Weight (kg)							
		Zone n (% of Total)	Within Range n (% of Zone)	Below Range n (% of Zone)	Above Range n (% of Zone)	Mean	SD	Range	Median
Gray	3-5 kg	25 (8.0%)	14 (56.0%)	6 (24.0%)	5 (20.0%)	4.60	1.73	2.57 to 9.16	4.45
Pink	6-7 kg	9 (2.9%)	5 (55.6%)	0 (0.0%)	4 (44.4%)	8.22	1.61	6.40 to 10.95	7.67
Red	8-9 kg	18 (5.8%)	8 (44.4%)	2 (11.0%)	8 (44.4%)	9.81	1.87	5.67 to 13.68	9.74
Purple	10-11 kg	48 (15.4%)	14 (29.2%)	17 (35.4%)	17 (35.4%)	11.04	1.63	7.74 to 14.00	11.06
Yellow	12-14 kg	39 (12.5%)	21 (53.8%)	7 (17.9%)	11 (28.2%)	13.72	2.33	9.67 to 20.00	13.10
White	15-18 kg	36 (11.5%)	15 (41.7%)	12 (33.3%)	9 (25.0%)	17.02	4.26	8.80 to 30.20	16.75
Blue	19-22 kg	18 (5.8%)	9 (50.0%)	3 (16.7%)	6 (33.3%)	22.44	5.00	15.40 to 32.30	20.25
Orange	23-29 kg	32 (10.3%)	13 (40.6%)	8 (25.0%)	11 (34.4%)	30.68	12.30	11.15 to 58.10	26.30
Green	30-36 kg	28 (9.0%)	12 (42.9%)	6 (21.4%)	10 (35.7%)	35.67	10.97	10.30 to 59.60	33.85
Black	37-49 kg	35 (11.2%)	12 (34.3%)	2 (5.7%)	21 (60.0%)	57.25	17.46	32.00 to 101.70	51.00

Abbreviations: ED, emergency department; EMS, Emergency Medical Services.

a For patients transported via providers using the Broselow method of weight estimation, if no Broselow color was included on the EMS chart, a Broselow color was assigned based on the weight estimation. Of 312 Broselow EMS runs, one had a prehospital weight of 1.4 kg (below Broselow gray), but an ED scale weight of 4.33kg. Another EMS run had a prehospital weight of 40.8kg but was documented as having a length above the Broselow black zone; ED scale weight was 77.4kg. Additionally, 22 EMS runs had a prehospital weight ≥50kg (above the Broselow black zone), but their ED scale weights ranged from 39 to 95.6kg (M=65.7kg, SD=13.5kg, Mdn=63.3kg).

*Handtevy—*Table 5 demonstrates the Handtevy-suggested weight estimation (based on age) compared to ED weights for patients of corresponding age included in this study; 40.7% of patients had estimated weights which placed them in the correct Handtevy “category” according to their ED weight.

**Table 5.** ED Scale Weights Compared to Handtevy Suggested Weights by Age ^a^

Age Group	ED Scale Weight (kg)					Handtevy Zone	Handtevy Predicted Weight	Percent Weight Discrepancy	
	n (%)	Mean	SD	Range	Median			Predicted vs Mean	Predicted vs Median
0-3.99 Months	40 (6.9%)	4.24	1.39	2.09 to 6.88	4.16	Gray	4 kg	−5.9%	−4.0%
4-5.99 Months	8 (1.4%)	7.03	1.27	4.85 to 9.16	7.08	Pink	6 kg	−17.2%	−18.0%
6-11.99 Months	42 (7.3%)	9.08	1.24	7.06 to 12.46	8.86	Red	8 kg	−13.4%	−10.8%
1-1.99 Years	106 (18.3%)	11.20	1.92	7.76 to 17.91	10.92	Purple	10 kg	−12.0%	−9.2%
2-2.99 Years	59 (10.2%)	14.75	2.93	10.10 to 24.50	14.00	Yellow	12 kg	−22.9%	−16.7%
3-3.99 Years	38 (6.6%)	17.37	6.40	11.20 to 52.40	16.00	White	15 kg	−15.8%	−6.7%
4-4.99 Years	24 (4.1%)	21.47	9.07	9.74 to 52.90	19.45	White	17 kg	−26.3%	−14.4%
5-5.99 Years	15 (2.6%)	24.39	7.05	15.90 to 43.20	21.70	Blue	20 kg	−22.0%	−8.5%
6-6.99 Years	23 (4.0%)	26.47	10.77	17.30 to 58.60	22.90	Blue	22 kg	−20.3%	−4.1%
7-7.99 Years	20 (3.5%)	30.85	9.51	16.80 to 52.90	28.05	Orange	25 kg	−23.4%	−12.2%
8-8.99 Years	23 (4.0%)	36.52	13.88	19.60 to 77.40	34.70	Orange	27 kg	−35.3%	−28.5%
9-9.99 Years	21 (3.6%)	39.01	12.15	17.40 to 68.60	38.10	Green	30 kg	−30.0%	−27.0%
10-10.99 Years	28 (4.8%)	44.52	12.80	18.10 to 80.20	45.30	Green	35 kg	−27.2%	−29.4%
11-11.99 Years	36 (6.2%)	53.33	17.17	28.90 to 95.60	53.80	Green	40 kg	−33.3%	−34.5%
12-12.99 Years	48 (8.3%)	60.22	17.21	32.60 to 101.70	59.25	Green	50 kg	−20.4%	−18.5%
13-13.99 Years	48 (8.3%)	62.47	19.00	39.10 to 122.00	58.50	Green	60 kg	−4.1%	2.5%

Abbreviation: ED, emergency department.

a Calculated as [(Predicted weight – mean ED scale weight)/Predicted weight]x100 or as [(Predicted weight – median ED scale weight)/Predicted weight]x100. Handtevy under-estimation when the value is negative, Handtevy over-estimation when the value is positive.

## Discussion

This study demonstrated no overall significant difference between the use of the Handtevy and Broselow methods with respect to the accuracy of prehospital weight estimation. This is in slight contrast to a systematic review by Young, et al who found that the Broselow method generally outperformed age-based formulas.^18^ Additionally, Lowe, et al compared theoretical Broselow and Handtevy (the length/age based edition) weight estimations based on National Health and Nutrition Examination Survey data and demonstrated increased accuracy of the Broselow tape, although the Handtevy system was more accurate for taller children.^12^ However, it is notable that many previous studies of weight estimation techniques have not examined their use in the prehospital care setting.^6,7,9–12,14,15,18,20^

Despite this, the weight estimation accuracy of the Broselow method in this study (51.3% of patient weights were within +/-10% of ED scale weights) was comparable to previous literature.^6,18^ Perhaps more importantly, EMS allocated 42.7% of patients to the appropriate Broselow category and 40.7% of patients to the appropriate Handtevy category. This is practically more relevant as medication dosing for pediatric patients is determined by weight category.

On average, the Broselow tape under-estimated patient weight by 6.9% and review of the literature demonstrates similar trends, although Both, et al examined data of over 3,000 patients and found that Broselow tended to over-estimate patient weight more frequently than under-estimate patient weight.^7,10,11^ Use of the Handtevy method was also associated with under-estimation of patient weight, on average approximately 6.3%. This is consistent with work by Lowe, et al who found that the Handtevy method tended to under-estimate patient weight.^12^

Based on the current study’s findings regarding weight under-estimation and documented concerns in the literature regarding the effect of the growing obesity epidemic on prehospital weight estimation, this study sample’s scale weight was further analyzed by age compared to the Handtevy application’s weight prediction.^14,15,18,20^ Overall, patients in this study weighed more than the Handtevy estimations, with the most profound differences occurring in Hispanic patients and those seven to eight years of age. It is not possible to suggest alterations to the Handtevy estimations or Broselow tape based on this study, as the dangers of over versus under dosing must be taken into account. However, it may be of some benefit for pediatric hospitals to study the average weights of their individual patient population and advise the relevant EMS providers accordingly.

Although the statistical analysis for this medication analysis was severely limited due to the small n, this study showed no significant difference between the use of the Handtevy or Broselow methods with respect to the medication dosing errors. The majority of medication errors in this study were due to incorrect weight estimation and a minority were due to EMS administering the incorrect dose for the estimated weight. Medication dosing error rates were quite high, but were not associated with a particular weight estimation method. Fifty percent of medications were inaccurately dosed. This is similar to the base rate found by Rappaport, et al, though well exceeds the rate of 34.7% reported by Hoyle, et al who studied records of over 5,000 children treated by paramedics.^4,22^ The increased error rates in this study may stem from differing definitions of medication error, the much smaller sample size in the current study, or weight estimation/provider error. The small sample size of the medication analysis is limiting, yet highlights the infrequent nature of EMS pediatric medication administration. In this study, weight-based medications were only given in 4.3% of EMS runs, very similar to the rate reported by Rappaport, et al (4.9%).^22^ This rarity in itself is a risk factor for dosing errors.^5^

## Limitations

A major limitation of this study is that the determination of prehospital weight estimation method was based entirely on EMS provider policy. It is possible that EMS providers may have used alternative methods which could confound the comparisons performed in this study and affect the results in unquantifiable ways. There are some data suggesting that EMS providers in this study, at least occasionally, used alternate methods; among patients where provider protocol dictated use of the Handtevy method, only 40.3% had documented prehospital weights corresponding to selections available on the Handtevy application. However, a comparison of estimation techniques still provides summative information, despite the inability to confirm fidelity to technique. Policy adherence will inevitably be a confounding variable, and real-world implementation data should be an important consideration for EMS with regards to policy decisions.

Additionally, this analysis was conducted just as Provider-Z transitioned from the Broselow method to the Handtevy method. This may have potentially led to errors due to unfamiliarity with the system. The small numbers of medications administered in this study may have led to Type 2 error. Other limitations include that since EMS estimated weights were analyzed as officially documented in the patient care report, EMS documentation errors are possible. Of note, although weights were compared to infant, standing, or bed scales regularly used in the ED, the accuracy of these scales was not verified outside of standard maintenance.

## Conclusion

This study demonstrated no statistically significant difference between the use of the Handtevy or Broselow methods with respect to either prehospital weight estimation or medication dosing. Overall, EMS tended to under-estimate patient weight, and actual weight tended to exceed Handtevy predictions. Further research is necessary in the prehospital care setting as this appears to be one of the first studies comparing these methods in practice. However, these results suggest similar field performance of the Broselow and Handtevy methods.
